# The p22 RNA Silencing Suppressor of the Crinivirus *Tomato chlorosis virus* is Dispensable for Local Viral Replication but Important for Counteracting an Antiviral RDR6-Mediated Response during Systemic Infection

**DOI:** 10.3390/v8070182

**Published:** 2016-06-28

**Authors:** Yazmín Landeo-Ríos, Jesús Navas-Castillo, Enrique Moriones, M. Carmen Cañizares

**Affiliations:** Instituto de Hortofruticultura Subtropical y Mediterránea “La Mayora”-Universidad de Málaga-Consejo Superior de Investigaciones Científicas (IHSM-UMA-CSIC), Estación Experimental “La Mayora”, 29750 Algarrobo-Costa, Málaga, Spain; landeoyazmin@gmail.com (Y.L.-R.); jnavas@eelm.csic.es (J.N.-C.); moriones@eelm.csic.es (E.M.)

**Keywords:** *Closteroviridae*, *Crinivirus*, *Tomato chlorosis virus*, RNA silencing suppressor, Infectious clone, Agroinoculation

## Abstract

Among the components of the RNA silencing pathway in plants, RNA-dependent RNA polymerases (RDRs) play fundamental roles in antiviral defence. Here, we demonstrate that the *Nicotiana benthamiana* RDR6 is involved in defence against the bipartite crinivirus (genus *Crinivirus*, family *Closteroviridae*) *Tomato chlorosis virus* (ToCV). Additionally, by producing a p22-deficient ToCV infectious mutant clone (ToCVΔp22), we studied the role of this viral suppressor of RNA silencing in viral infection in both wild-type and RDR6-silenced *N. benthamiana* (NbRDR6i) plants. We demonstrate that p22 is dispensable for the replication of ToCV, where RDR6 appears not to have any effect. Furthermore, the finding that ToCV∆p22 systemic accumulation was impaired in wild-type *N. benthamiana* but not in NbRDR6i plants suggests a role for p22 in counteracting an RDR6-mediated antiviral response of the plant during systemic infection.

In plants, RNA silencing is a conserved sequence-specific RNA-mediated mechanism of gene regulation that also serves as an antiviral defence [1]. This mechanism is triggered by double-stranded RNA (dsRNA), which in single-stranded RNA (ssRNA) viruses can derive from highly structured ssRNAs or be generated during their replication [2]. These dsRNAs are processed by RNase III-type Dicer-like (DCL) enzymes into small RNAs (sRNAs) [3] that are incorporated into the RNA-induced silencing complex, leading to sequence-specific degradation of target RNAs. This process is amplified by host-encoded RNA-dependent RNA polymerases (RDRs) that convert single-stranded RNA into dsRNA, which is subsequently processed by DCL into secondary sRNAs. Among the plant RDRs implicated in virus defence, RDR6 plays a relevant role [4,5,6]. RDR6 is involved in short-, long-range cell-to-cell and long-distance signalling of RNA silencing [7,8]. The involvement of RDR6 in virus defence is supported by the fact that the downregulation of *RDR6* through RNA interference in *Nicotiana benthamiana* plants (RDR6i plants) can result in hypersusceptibility to some viruses [5,6].

To counteract antiviral RNA silencing, most plant viruses express viral suppressors of RNA silencing (VSRs) that act at different steps in the silencing pathway to facilitate the infection process [9]. Therefore, to achieve a successful plant invasion, viruses must replicate and move both cell-to-cell and systemically to distant parts of the plants, confronting the RNA silencing defence responses of the plant at each of these steps. The use of suppressor-deficient viruses has been an informative approach for studying the VSR role during these steps of viral infection.

*Tomato chlorosis virus* (ToCV, genus *Crinivirus*) belongs to the family *Closteroviridae*, in which the largest RNA genomes among plant viruses have been reported [10]. ToCV causes a yellow leaf disorder syndrome that primarily affects tomato (*Solanum lycopersicum*) and is transmitted in nature by the whiteflies (Hemiptera: Aleyrodidae) *Bemisia tabaci*, *Trialeurodes vaporariorum* and *T. abutiloneus* [11]. ToCV has a bipartite single stranded, positive-sense RNA genome with RNA1 encoding proteins involved in viral replication and RNA2 encoding proteins involved in encapsidation, movement, and whitefly transmission [12,13,14,15]. Although both RNAs are required for the effective infection of plants, RNA1 can replicate independently, whereas RNA2 depends on RNA1 for its replication [16]. Similar to other members of the family *Closteroviridae* [17,18], ToCV encodes multiple VSRs, the major coat protein (CP) and minor coat protein (CPm) in RNA2, and the p22 protein located at the 3’-proximal genomic region of RNA1 [19]. It has been shown that p22 very efficiently suppresses local RNA silencing induced either by sense RNA or by dsRNA. Although VSRs exhibit broad ranges of structural diversity and modes of action, in the family *Closteroviridae*, counterparts of genes encoding VSRs are found at the 3’-end of the monopartite genomes of viruses in the genera *Closterovirus* and *Ampelovirus* and at the 3’ end of RNA1 in viruses of the mostly bipartite genus *Crinivirus* [10]. It is worth mentioning that some of the VSRs were shown to enhance viral infection before being identified as suppressors [9]. Thus, by deletion analysis of the 3’-end genes of the closterovirus *Citrus tristeza virus* (CTV), it was found that the p23 VSR controls the asymmetric accumulation of CTV RNAs by downregulating negative-stranded RNA accumulation and indirectly increases the expression of 3’ genes [20]. The p21 VSR encoded in the 3’-end of the genome of the closterovirus *Beet yellow virus* (BYV) [21] was previously identified as an enhancer of RNA amplification [22]. In the case of the crinivirus *Lettuce infectious yellows virus* (LIYV), although the 3’-end RNA1-encoded p34 protein has not been described as a VSR, it enhances the trans-replication of RNA2 [23].

Understanding the underlying mechanisms in plant virus confrontations is a prerequisite for control of viral diseases. In this work, we demonstrate that RDR6 plays an antiviral role in plant defence against ToCV. Additionally, by using a p22-deficient ToCV infectious mutant clone (ToCVΔp22), we investigated the specific role of this protein in the viral infection process in both wild-type and RDR6-silenced *N. benthamiana* plants.

To analyse whether NbRDR6 plays a role in plant defence against ToCV, virus accumulation was studied in the *N. benthamiana* line RDR6i, in which NbRDR6 is constitutively silenced [6]. To this end, whitefly-mediated inoculation assays were conducted within insect-proof cages using individuals of the *Bemisia tabaci* Mediterranean species (MED) (formerly biotype Q) from a healthy population reared on melon (*Cucumis melo* cv. ANC 42, La Mayora-CSIC seed bank). Viruliferous whiteflies were obtained by allowing adult individuals a 48-h acquisition access period (AAP) on ToCV-infected tomato plants (cv. Moneymaker). After AAP, viruliferous whiteflies were transferred to healthy wild-type or RDR6i *N. benthamiana* plants (3 to 5 leaf stage) (fifty individuals per plant) for a 48-h inoculation access period using clip-on cages. Virus accumulation levels were estimated by quantitative real-time reverse transcription-polymerase chain reaction (qRT-PCR). Total RNA was extracted as described previously [24] from pools of young non-inoculated leaves from three infected plants per treatment at 25 days post-inoculation. RNA was treated with Turbo DNase (Ambion, Austin, TX, USA) and quantified. Total RNA (300 ng) was reverse transcribed with specific primers using AMV RT (Promega, Madison, WI, USA). For qRT-PCR, cDNA was used in 20 µL reactions with SYBR *Premix Ex Taq* (Perfect Real Time) (TaKaRa Biotech, Dalian, China) according to the manufacturer’s instructions. Three technical replicates were performed per cDNA of each biological sample. Primers for the ToCV RNA2 viral sequence (MA1178 and MA1179) and for the conserved Solanaceae gene clathrin adaptor complex AP-2 (CAC) (MA1279 and MA1280) as a housekeeping internal standard [25] were designed to amplify fragments of similar size. The sequences of the primers used are listed in Supplementary Table S1. The relative quantification of viral RNA was calculated using the 2^−∆∆C(t)^ method [26]. The results revealed an approximately 4-fold increase in ToCV RNA accumulation in RDR6i compared with wild-type plants infected with the virus (Figure 1A). Although this increased susceptibility did not always correlate with an enhancement of the disease symptoms, an increase in the interveinal chlorosis in older leaves, which is typical of ToCV infection in tomato, was observed in some RDR6i plants (exemplified in Figure 1B). Therefore, these results indicate that the reduced RDR6 expression in *N. benthamiana* plants resulted in increased ToCV susceptibility, suggesting that RDR6 plays a relevant role in antiviral defence.

Considering that RDR6 is a component of the RNA silencing pathway in plants, we presumed that a VSR could be involved in counteracting this antiviral response. We assessed the role of the VSR p22 of ToCV during the infection process by using a p22-deficient ToCV mutant clone created by deleting the complete p22 gene sequence. To obtain the construct ToCV-RNA1∆p22, we used the ToCV RNA1 infectious clone p35S-ToCV-RNA1 [16], that harbours two *PstI* sites, one at position 6911 and the other at position 8614. The deletion mutant was generated by PCR-driven overlap extension [27], amplifying two overlapping DNA fragments in separate PCRs. In the first PCR, a 763-bp DNA fragment containing the *Pst*I site of ToCV RNA1 at position 6911 was amplified using the primer pair MA1543/MA1544. In a second PCR, a 395-bp DNA fragment containing the *Pst*I site of ToCV RNA1 genome at position 8614 was amplified using the primer pair MA1545/MA1546. These initial PCRs resulted in overlapping segments containing part of the flanking sequences of the p22 genomic region, which were mixed and amplified in a third PCR with primers MA 1543 and MA 1546 to produce a 1132-bp chimeric fragment with a deleted p22. The ToCV RNA1 p22 deletion mutant was constructed by exchanging the *Pst*I restriction fragment of the p35S-ToCV-RNA1 infectious clone with the chimeric fragment amplified in the third PCR indicated above, which was also obtained by digestion with *Pst*I (Supplementary Figure S1). The p22-deficient construct was then cloned into *E. coli* and introduced into *Agrobacterium tumefaciens* cells (strain GV3101). The nucleotide sequences of the primers used are provided in Supplementary Table S1.

To ensure that both the wild-type and the p22-deficient mutant constructs were efficiently and equally transcribed *in vivo* from the 35S promoter, a preliminary agroinfiltration analysis was performed in *N. benthamiana* plants after short periods post-infiltration (3 and 5 days post-infiltration (dpi)). Thus, plants (3–5 leaf stage) were agroinfiltrated with *A. tumefaciens* GV3101 carrying ToCV-RNA1 or ToCV-RNA1∆p22 clones at a OD_600_ of 1, as previously described [16]. For Northern blot analysis, total RNA was extracted from agroinfiltrated patches as described above, and viral RNA was detected using digoxigenin (DIG)-labelled positive and negative sense-specific ssRNA probes for the RNA1 3’-end as described previously [16]. The analysis of positive strands at 3 dpi exhibited similar accumulation levels of transcripts from both the ToCV-RNA1 and ToCV-RNA1∆p22 constructs (Figure 2A). Therefore, the clear differences in the RNA accumulation levels of the positive strands observed at 5 dpi for the two constructs indicated differences in local replication (Figure 2A). A time-course analysis of the local replication levels of wild-type ToCV and ToCVΔp22 was subsequently conducted at 5 and 7 dpi. We compared the viral accumulation levels in patches agroinfiltrated with infectious clones of ToCV-RNA1 or ToCV-RNA1∆p22 in combination with the infectious clone of ToCV-RNA2 in both wild-type and RDR6i *N. benthamiana* plants. In the agroinfiltrated leaves of wild-type plants, the accumulation of both positive and negative viral RNA strands of ToCV-RNA1∆p22 were increased compared to that of ToCV-RNA1 (Figure 2B). Therefore, we hypothesized that the loss of certain secondary structure elements in the deletion construct could facilitate the replication process. Indeed, by using the MFOLD algorithm [28] we found that the presence of the p22 sequence leads to an increase in the number of potential stem loop structures (Supplementary Figure S2). Interestingly, the absence of the p22 gene sequence resulted in an increased accumulation of negative strand molecules at both 5 and 7 dpi (Figure 2B). To assess whether p22 plays a role in the trans-accumulation of ToCV-RNA2, the patches were analysed by Northern blot analysis using a DIG-labelled specific RNA probe for the coat protein (CP) gene [16]. In this case, a band corresponding to the genomic RNA2 was observed in all cases, and it was more intense in co-infiltrations of the wild-type ToCV RNA1 with ToCV RNA2 (Figure 2B). Although the viral accumulation levels of ToCV-RNA1∆p22 were consistently increased compared with ToCV-RNA1, no increased accumulation of RNA2 was observed in these cases. Similar results were obtained for agroinfiltrated leaves of NbRDR6i plants (Figure 2B), which indicated that it is unlikely that NbRDR6 influences local ToCV replication as described for PVX [6]. These results were independently reproduced twice. Overall, the previous results indicated that p22 is not required for the local replication of ToCV-RNA1 but does appear to influence the trans-accumulation of ToCV-RNA2.

After evaluating the role of p22 in local replication, we studied its role during systemic ToCV infection of plants. For this purpose, we conducted two independent experiments in which wild-type and RDR6i plants were co-infiltrated with a mixture of *A. tumefaciens* containing ToCV-RNA1 or ToCV-RNA1∆p22 and ToCV-RNA2 (15 wild-type and 15 RDR6i plants per combination and experiment). The number of systemically infected plants was determined by tissue blot hybridization of petiole sections of upper non-infiltrated leaves at 30 dpi, using a probe specific for the CP gene [16]. The effects of viruses and genotypes were analysed by applying generalized linear models (GzLM) (IBM SPSS Statistics v. 22 software) in which pair-wise comparisons were performed using the sequential Bonferroni method for error correction. Data sets were expressed as the numbers of infected and non-infected plants and analysed by GzLM using Logit as the link function and Binomial as the underlying distribution. Thus, it was observed that ToCV∆p22 was unable to support an efficient systemic infection in wild-type *N. benthamiana* plants since significantly lower number of plants (*p* = 0.025) resulted infected compared to the infections with ToCV (compare ToCV and ToCV∆p22 in Figure 3). Therefore, these results indicated that the presence of p22 is important to support the efficient systemic infection of wild-type *N. benthamiana* plants. In contrast, in RDR6i plants, both ToCV and ToCV∆p22 exhibited similar systemic infection ability since no significant differences (*p* = 0.89) in the number of infected plants in the absence or presence of p22 were found (Figure 3). These results indicated that for ToCV∆p22, the ability to systemically infect plants was rescued in RDR6i plants. Interestingly, the absence of RDR6 in RDR6i plants resulted in an enhancement of the leaf chlorotic symptoms in infected plants for both ToCV and ToCV∆p22. Altogether, these results suggest a role of p22 in counteracting an antiviral RDR6-mediated response during the process of systemic infection.

In this work, we demonstrated that RDR6 plays a relevant role in controlling ToCV accumulation in plants. Our results also indicate that NbRDR6 does not influence local ToCV replication either in the presence or absence of p22 protein. Although p22 does not appear to be necessary for local RNA1 replication, it may influence the equilibrium between the positive and negative strands of the virus during the infection process. Therefore, it was noticeable that a clear accumulation of negative strands of ToCV RNA1∆p22 occurred in the absence of p22 (Figure 2B). Similarly, the absence of the p23 VSR at the 3’-end of the genome of the closterovirus CTV was associated with a substantial increase in the accumulation of negative-stranded RNAs, especially those corresponding to subgenomic RNAs (sgRNA) [20]. Apparently, this increased accumulation of negative-stranded sgRNA reduced the availability of the corresponding positive-stranded sgRNA as a messenger. In the case of ToCV, the increase in negative strands of ToCV RNA1∆p22, which might result in increased accumulation of positive strands from which the replication associated proteins are translated, does not correlate with increased RNA2 accumulation. We hypothesize that, as described for CTV, the increase in negative strands in the absence of p22 affects the availability of positive strands as messengers, leading to a reduction in products translated from RNA1, where the replicase is encoded. Alternatively, as the replication of RNA2 is delayed compared with that of RNA1 [16], the presence of p22 might protect the RNA2 template that has to be replicated. This could also explain the increase of the RNA2 accumulation levels observed in co-infiltrations with wild-type ToCV RNA1. Interestingly, mutagenesis studies of the crinivirus LIYV RNA1 3’-end ORF, encoding P34, showed that although the mutations introduced did not affect the replication of the LIYV RNA1, they reduced the accumulation of LIYV RNA2 [23]. Significantly, we demonstrated that the p22 VSR appears to be important for the successful ToCV systemic infection of plants, counteracting an antiviral RDR6-mediated response. Similarly, RDR6 restricts systemic infection by the VSR-defective viruses *Cucumber mosaic virus* (CMV) and *Turnip mosaic virus* (TuMV) [29,30,31].

The results shown in this work, together with the results obtained for the p22 protein when isolated from the viral context that demonstrate that p22 preferentially binds long dsRNAs [32], have allowed us to propose a model for the RDR6-p22 confrontation during ToCV infection. Thus, after the induction of antiviral silencing in the recipient tissues, the new dsRNA synthesized by RDR6 is processed into the secondary sRNA that targets the viral RNA for degradation. When ToCV produces p22, this protein would bind to the long dsRNAs generated by RDR6, avoiding their cleavage in sRNA and thus interfering in the progression of the silencing process. When p22 is absent (ToCV∆p22), the virus is silenced more efficiently, as RDR6 generates the long dsRNAs that are processed into sRNAs to target ToCV∆p22, impairing the systemic spread of the virus. In RDR6i plants in which RDR6 is constitutively silenced, efficient ToCV∆p22 infections occur.

In summary, the use of a p22 VSR-deficient version of ToCV allowed us to advance our understanding of the ToCV infection process, a necessary preliminary step in addressing this important viral disease.

## Figures and Tables

**Figure 1:**
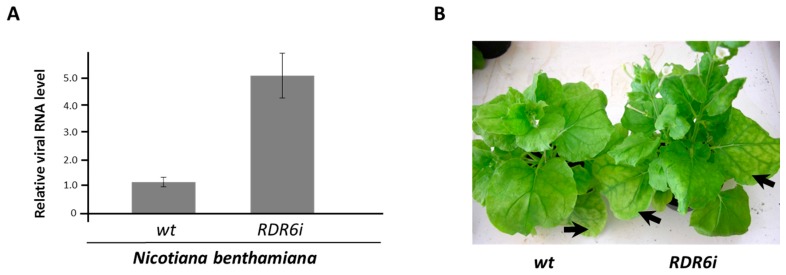
Enhanced susceptibility to *Tomato chlorosis virus* (ToCV) transmitted using its natural vector *Bemisia tabaci* in *Nicotiana benthamiana* RDR6i plants at 25 days post-infiltration. (**A**) Quantitative real-time RT-PCR of ToCV RNA2 in infected *N. benthamiana* wild-type and RDR6i plants performed using pools of three plants each. Error bars represent standard deviation for three replicates. Values are relative to levels detected in wild-type plants, which were given an arbitrary value of 1; (**B**) Interveinal chlorotic symptoms of ToCV in infected *N. benthamiana* wild-type and RDR6i plants. Leaves exhibiting enhanced interveinal symptoms are indicated by arrows.

**Figure 2:**
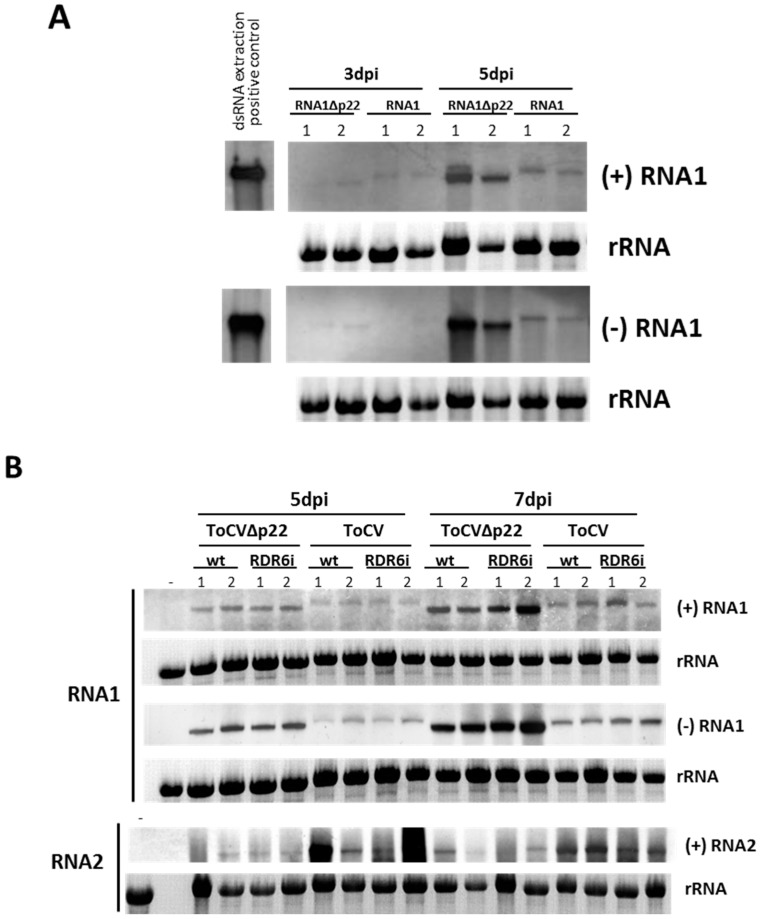
Northern blot analysis using digoxigenin (DIG)-labelled RNA probes to detect the positive (+) or negative (−) sense RNA forms during local infection with wild-type or p22 mutant *Tomato chlorosis virus* (ToCV) (ToCV and ToCVΔp22, respectively). (**A**) Local RNA1 replication and accumulation of (+) and (−) sense strands [(+) RNA1 and (−) RNA1] at 3 and 5 dpi in patches of wild-type *Nicotiana benthamiana* agroinfiltrated with RNA1 infectious clones of ToCV or ToCVΔp22. As a positive control, a dsRNA extraction from a ToCV infected plant was used; (**B**) Local replication and accumulation of (+) and (−) sense strands [(+) RNA1 and (−) RNA1] of RNA1, and accumulation of (+) strand [(+) RNA2] of RNA2 at 5 and 7 dpi in patches of wild-type and RDR6i *N. benthamiana* plants agroinfiltrated with infectious clones of RNA1 of ToCV or ToCVΔp22 and RNA 2 of ToCV. Samples from two independent plants (1,2) were analysed. Ethidium bromide-stained rRNA was used as loading control.

**Figure 3:**
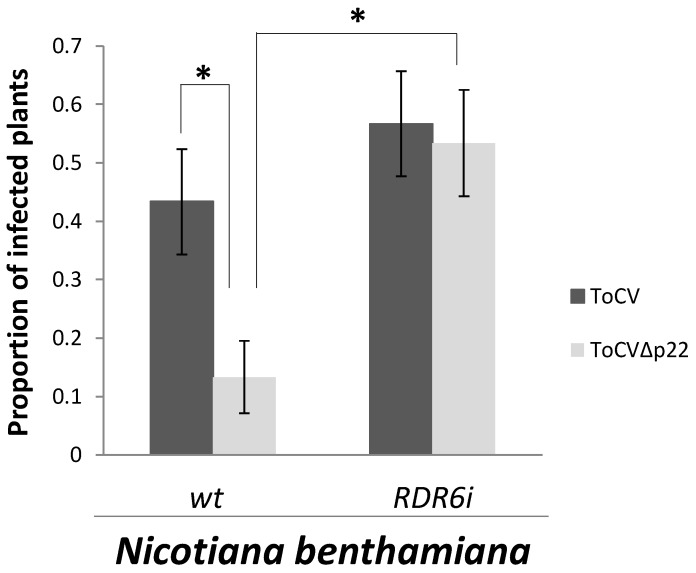
Systemic infection rate (mean ± standard error (SE)) of *N. benthamiana* wild-type and *RDR6i* plants inoculated with infectious clones of RNA1 of ToCV or ToCVΔp22 and RNA2 of ToCV from two independent experiments. Infections of ToCV and ToCVΔp22 were compared by the generalized mixed linear model with binomial error structure. For each genotype, infections with both viruses were compared by sequential Bonferroni tests. Bars represent the standard error of the mean, and asterisks indicate significant differences (*p* < 0.05).

## Supplementary Materials

The following are available online at www.mdpi.com/1999-4915/8/7/182/s1, Figure S1: Diagram of the methodology used to create the p22-deficient ToCV RNA1 mutant clone, Figure S2: Lowest free energy secondary structure prediction of 3’-ends of the coding strand of ToCV RNA1, Table S1: Nucleotide sequences of primers used in this work.
