# Supplementary material for: The p22 RNA Silencing Suppressor of the Crinivirus Tomato chlorosis virus is Dispensable for Local Viral Replication but Important for Counteracting an Antiviral RDR6-Mediated Response during Systemic Infection
Source: Viruses. 2016 Jun 28;8(7):182. doi: 10.3390/v8070182 (PMC4974517; doi:10.3390/v8070182)
Supplement: Supplementary file 1 [file viruses-08-00182-s001.pdf]

Supplementary Materials: The p22 RNA Silencing Suppressor of the Crinivirus *Tomato chlorosis virus* is Dispensable for Local Viral Replication but Important for Counteracting an Antiviral RDR6-Mediated Response during Systemic Infection

Yazmín Landeo-Ríos, Jesús Navas-Castillo, Enrique Moriones and M. Carmen Cañizares *

Table S1. Nucleotide sequences of primers used in this work.

Gene/cDNA Clone	Primer		Primer Sequence (5′–3′) [1]	Primer Position [1,2]
ToCV-RNA1 clone	MA 1543	(FW)	AACTGCAGAAGCCATTGACA	6907–6926
	MA 1544	(RV)	***TTGAATATTAACCAGA***/**ACTGACCTAAAT**AACAACAGTC	8259–8244/7661–7640
	MA 1545	(FW)	***ATTTAGGTCAGT***/**TCTGGTTAATATTCAA**AGGATGG	7650–7661/8244–8266
	MA 1546	(RV)	GATCTGCAGGTCGACTCTAG	8620–8601
ToCV-RNA2 clone	MA 1178	(FW)	ACCGGGCGCAGTTCATACAA	1522–1541
	MA 1179	(RV)	CCGACAAGAAACAGCGCTCC	1697–1675
CAC	MA 1279	(FW)	CTCCGTTGTGATGTAACTGG	779–798
	MA 1280	(RV)	ATTGGTGGAAAGTAACATCATC	950–929

[1] The underlined sequences are restriction endonucleases sites introduced into the primers for cloning (*Pst* I in MA 1543 and MA 1546). Nucleotides in bold indicate overlapping sequences. Nucleotide sequences in italics and positions separated with the slash symbol (/) indicate the p22 flanking sequences. FW and RV indicate forward and reverse sense primers, respectively; [2] Primer positions indicate the positions in the RNA1 and RNA2 nucleotide sequences of AT80/99 ToCV isolate (GenBank accession numbers DQ983480 and DQ136146, respectively) and the CAC gene of Solanaceae (GenBank accession number XM_009783969.1) used as internal standard.

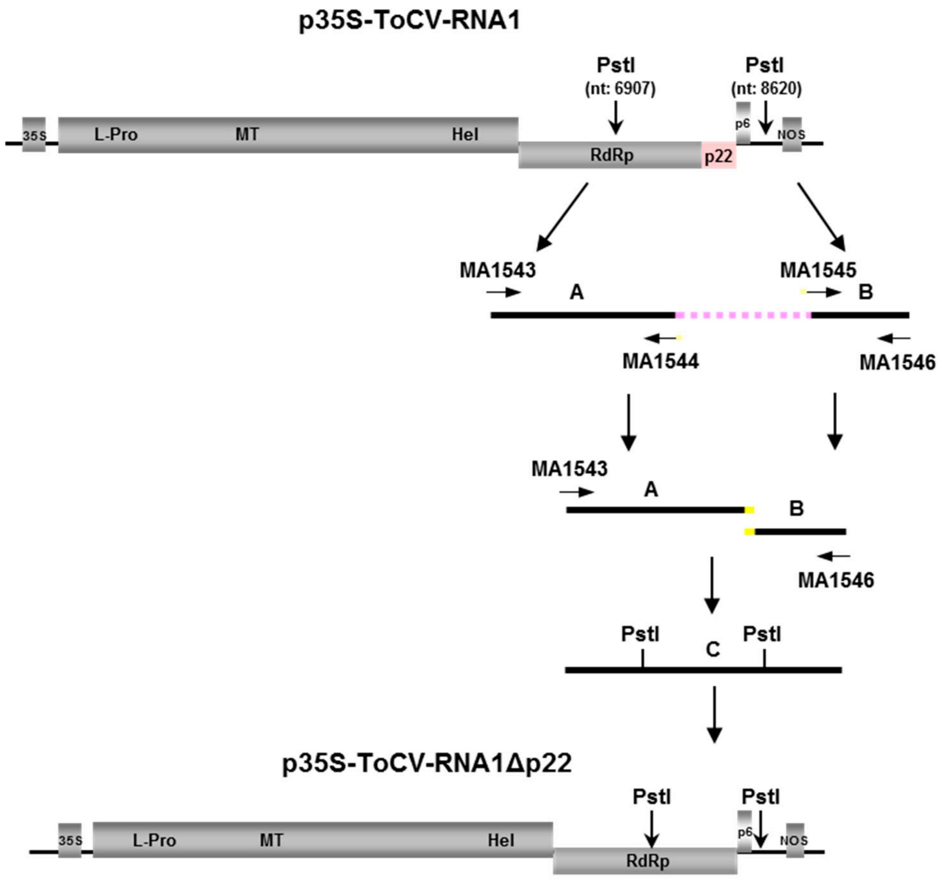

Figure S1: Diagram of the methodology used to create the p22-deficient ToCV RNA1 mutant clone.

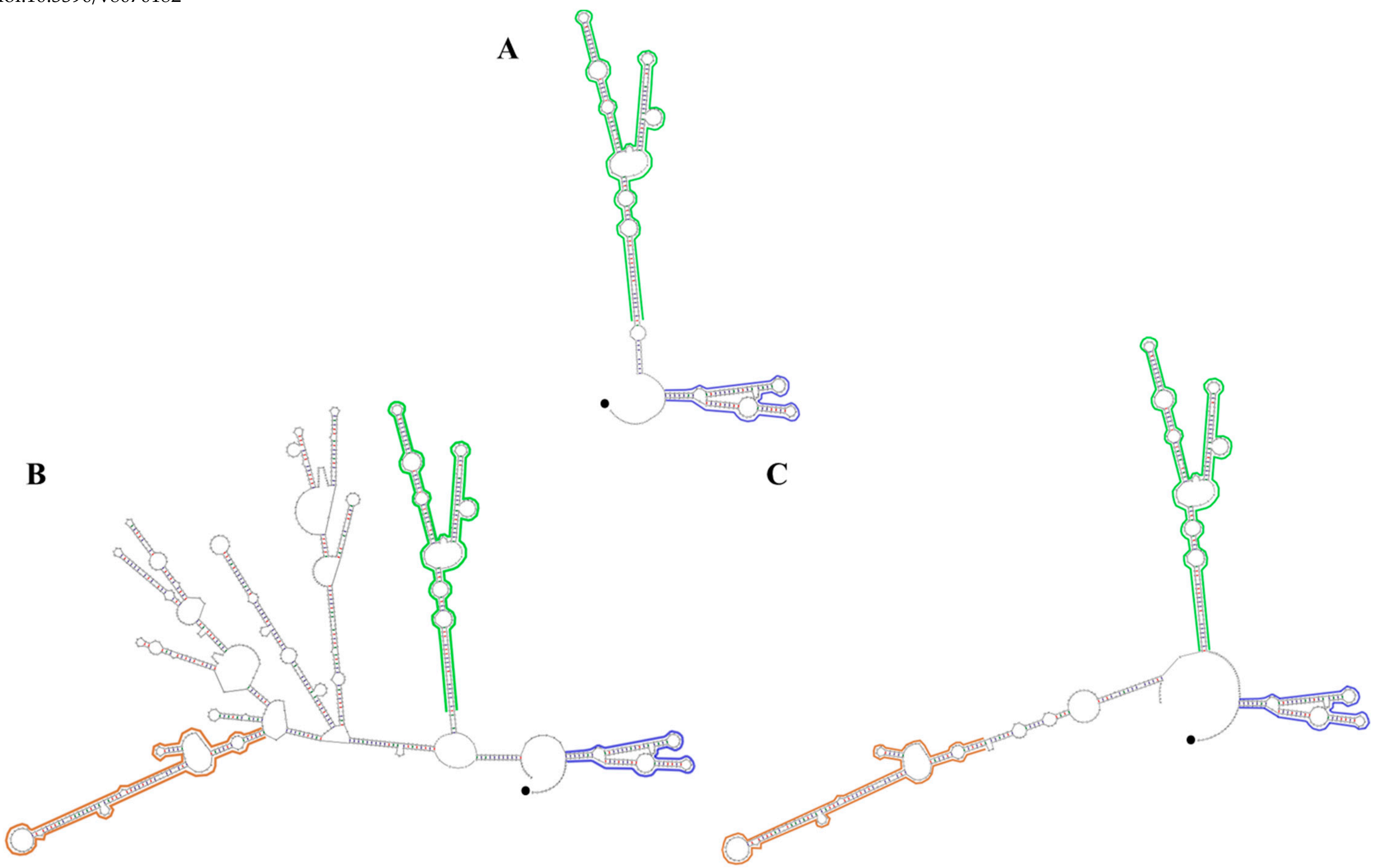

Figure S2. Lowest free energy secondary structure prediction of 3′-ends of the coding strand of ToCV RNA1. (**A**) Predicted secondary structure of the entire 3′-UTR (nt: 8237-8594); (**B**) Predicted secondary structure of the 3′-end comprising the 3′-UTR, the p22 sequence and the 241 nt located before the p22 sequence (nt: 7421-8594); (**C**) Predicted secondary structure of the 3′-end comprising the 3′-UTR and the 241 nt located before the p22 sequence (nt: 7421-7662 and 8237-8594). Identical stem loops are highlighted with similar colors. The 3′-end of each structure is indicated with (●). The RNAs were folded using the mfold program (Zuker, 2003).

© 2016 by the authors. Submitted for possible open access publication under the terms and conditions of the Creative Commons Attribution (CC-BY) license (http://creativecommons.org/licenses/by/4.0/).
